# Experimental infection of Rio Mamore hantavirus in *Sigmodontinae* rodents

**DOI:** 10.1590/0074-02760160021

**Published:** 2016-06

**Authors:** William Marciel de Souza, Alex Martins Machado, Luiz Tadeu Moraes Figueiredo

**Affiliations:** 1 Universidade de São Paulo, Faculdade de Medicina de Ribeirão Preto, Centro de Pesquisa em Virologia, Ribeirão Preto, SP, Brasil; 2 Universidade Federal de Mato Grosso do Sul, Três Lagoas, MS, Brasil

**Keywords:** experimental infection, Rio Mamore hantavirus, wild *Sigmodontinae* rodents

## Abstract

This study shows an experimental spillover infection of*Sigmodontinae* rodents with Rio Mamore hantavirus (RIOMV).*Necromys lasiurus* and *Akodon sp* were infected with 10^3^ RNA copies of RIOMV by intraperitoneal administration. The viral genome was detected in heart, lung, and kidney tissues 18 days after infection (ai), and viral excretion in urine and faeces began at four and six ai, respectively. These results reveal that urine and faeces of infected rodents contain the virus for at least 18 days. It is possible that inhaled aerosols of these excreta could transmit hantavirus to humans and other animals.

The hantavirus is a genus in the *Bunyaviridae* family, and its genome includes three segments of single-stranded negative-sense RNA, termed L (large), M (medium) and S (small). The L segment encodes the viral RNA polymerase, the M segment encodes two envelope glycoproteins (Gn and Gc), and the S segment encodes the viral nucleocapsid protein (N). The hantavirus may also have a small nonstructural protein (NS) that was previously described in Tula, Puumala and Andes viruses (Jaaskelainen et al. 2007, Jonsson et al. 2010, Vera-Otarola et al. 2012, Vaheri et al. 2013).

Human pathologies caused by the hantavirus are divided into two severe forms: haemorrhagic fever with renal syndrome (HFRS) in Asia and Europe and hantavirus cardiopulmonary syndrome (HCPS) in the Americas (Clement et al. 2012). Since 1993, approximately 4,000 cases of HCPS have been reported in the Americas, and these cases were found to be caused by more than 30 hantavirus strains or genetic lineages (Souza & Figueiredo 2014). Almost half of these HCPS cases occurred in Brazil, where the disease is often seasonal and produces a high case fatality rate that reaches 50% among patients infected with Araraquara virus (ARAQV) (Figueiredo et al. 2014).

Hantaviruses cause persistent infections in rodents, but the kinetics of this infection in *Sigmodontinae* and the period of viral excretion in the host’s excreta are not clearly defined. Additionally, infected rodents that are not natural reservoirs (spillover events) of a given hantavirus can potentially contribute to the evolution and maintenance of the pathogen in nature (Jonsson et al. 2010). While we note that it is essential to understand the mechanism by which hantaviruses are transmitted among rodents and humans, our study aims to evaluate the kinetics of infection and the period of shedding of Rio Mamore hantavirus (RIOMV) in the excreta of *Sigmodontinae* rodents.

The rodents were captured by Sherman traps in Ribeirão Preto, São Paulo, Brazil. The trapped animals were anesthetised and identified by morphological characteristics. Blood was then collected by puncturing the retro-orbital venous plexus and submitted for detection of IgG antibodies by ELISA, using recombinant nucleoprotein of ARAQV (rN-ARAV) as the antigen, and for RT-PCR analysis, both as previously described (Moreli et al. 2004, Padula et al. 2004, Figueiredo et al. 2008, 2009a).

*Sigmodontinae* rodents that were not previously infected by hantavirus were maintained in quarantine conditions. Then, the rodents were transferred to a biosafety level-3 (BSL-3) laboratory/vivarium (Jonsson et al. 2010) and maintained under standard laboratory conditions (12/12 h light-dark cycle on at 11:00/off at 22 ± 2ºC; food and water provided *ad libitum*).

All procedures for the capturing and handling of wild animals were authorised by Instituto Brasileiro do Meio Ambiente e dos Recursos Naturais Renováveis - IBAMA (0115/07 SUPESP/Fauna/LIC) and by the Ethics Committee on Animal Experiments of the Universidade de São Paulo in Ribeirão Preto City School of Medicine (113/2006).

The rodents were infected with the RIOMV strain HTN-0007. The virus was cultivated for 14 days in Vero E6 cells (African green monkey kidney cells) (de Pádua et al. 2015). Culture supernatants were collected from the flasks of infected cells and aliquoted for use as the viral stock.

The RNA of the viral stock as well as that of the mouse samples was extracted using the QIAamp viral RNA extraction kit (Qiagen, Hilden, Germany) according to the manufacturer’s protocol. Then, for detection and quantification of RIOMV in the viral stock and in the mouse samples, we used a one-step SYBR Green I real-time RT-PCR assay (Machado et al. 2013). RT-PCR was performed in triplicate using the StepOnePlus Real-Time PCR System (Applied Biosystems, Foster City, CA, USA), the SuperScript III Platinum SYBR Green One-Step Kit (Invitrogen, Carlsbad, CA, USA), and hantavirus primers that amplify 264 base pairs (bp) of the N gene in the small RNA segment of RIOMV, as previously reported (Moreli et al. 2004).

After RT-PCR measurements, the concentration of transcribed RNA extract from the RIOMV stock, in copies/μL, was converted to copy number using the following formula: RNA copy number (copies/μL) = (RNA concentration (g/μL)/number of nucleotides of transcript x 340) x 6.022x10^23^. ΔRn amplification results were obtained from ten-fold serial dilutions of the transcribed RNA by real-time RT-PCR and were used to create a standard curve with a known number of RIOMV RNA copies of per mL, as previously described (Machado et al. 2013).

For the rodent experiments, *Sigmodontinae* animals free of hantavirus infection were divided into two groups of three animals each, both including one*Necromys lasiurus* animal and two *Akodon sp*animals. The animals were anesthetised in a glove box by intraperitoneal injection of 7 mg/kg of Xylazine and 100 mg/kg of Ketamine. Rodents in the first group were intraperitoneally administered 200 μL containing 10^3^ RNA copies of RIOMV. Animals in the second group were used as negative controls and received intraperitoneal administration of 200 mL of culture supernatant from uninfected cells. The animals were housed individually in metabolic cages, and their urine, faeces and oropharyngeal secretions (obtained using sterile cotton swabs) were collected daily for 18 days. Blood was collected from the retro-orbital venous plexus at 9, 14 and 18 days after infection (ai). On day 18, the rodents were sacrificed by intraperitoneal injection of 16 mg/kg of Xylazine and 120 mg/kg of Ketamine, and their salivary glands, lungs, hearts, spleens, livers and kidneys were collected. All biological samples were subjected to real-time RT-PCR for RIOMV, as described above.

Based on the collected blood samples, all rodents infected with RIOMV presented viremia by the 9th day ai, and the virus was still detected by the 18th day ai, with a slight decrease in the viral load at this later time point (Figure). Tissues from salivary glands, lungs, hearts, spleens, livers and kidneys of the infected rodents were all tested by real-time RT-PCR for RIOMV, as described above. The organs of the rodents were collected 18 days ai, and they were all found to contain RIOMV particles, excluding the salivary glands, as shown in Table. The viral loads were as follows: 2.3 to 4.3 x 10^3^ RNA copies/ml in the lungs, 2.2 to 3.3 x 10^3^ RNA copies/ml in the hearts and 2.1 to 2.6 x 10^2^ RNA copies/mL in the kidneys. Interestingly, spleen and liver RIOMV particles were detected in only one animal each at low viral loads (0.8 and 1.2 x 10^3^ RNA copies/mL, respectively). As expected, blood, organs, faeces or urine isolated from animals in the negative control group did not show hantavirus genome amplification. The excretion of RIOMV in the urine of infected animals began at the 4th day ai with a viral load of 10^4^ copies of RNA per mL. The RIOMV load increased on the 5th day ai and remained at a plateau until the 18th day ai (Figure). The excretion of RIOMV in faeces was first observed on the 6th day ai, with 10^4^ copies of RNA per mL, and progressively increased until the 8th day ai, at which point it plateaued with only small variations until the 18th day ai (Figure).

**Figure:**
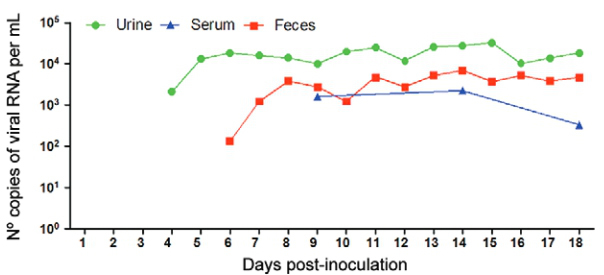
Kinetics of Rio Mamore viremia and viral excretion in faeces and urine of Sigmodontinae rodents monitored for 18 days post-infection.

**TABLE:** Viral loads in organs of *Sigmodontinae* rodents 18 days after intraperitoneal infection with Rio Mamore virus

Organs	Rodent Nº 1	Rodent Nº 2	Rodent Nº 3
Salivary gland	Not detected	Not detected	Not detected
Lung	4.3x10^3^	2.7x10^3^	3.1x10^3^
Heart	3.3x10^3^	2.2x10^3^	2.8x10^3^
Spleen	Not detected	1.2x10^3^	Not detected
Liver	0.8x10^3^	Not detected	Not detected
Kidney	2.6x10^2^	2.1x10^2^	2.3x10^2^

Viral load is expressed in RNA copies per mL. Not detected means samples where the viral genome was not amplified.

A hallmark of hantaviruses is their ability to establish chronic infections in their primary rodent hosts. The factors contributing to hantavirus persistence in rodents are not clearly understood, but it is known that these infections are marked by a short acute stage that produces high levels of infectious virus, followed by a prolonged chronic infection wherein the virus is usually found at much lower levels (Padula et al. 2004).

In our study, we have shown that RIOMV was able to infect *N. lasiurus*and *Akodon sp*, both *Sigmodontinae* species that have not been reported as natural reservoirs for RIOMV. The animals excreted hantavirus particles in their urine and faeces at least 18 days ai. Excretion levels of RIOMV were similar in both infected rodent species, suggesting that their urine and faeces could transmit the virus to other animals, including humans. Our results corroborate those observed in other *Sigmodontinae* species infected with other hantaviruses, including *Oligoryzomys longicaudatus* experimentally infected by Andes virus and *Peromyscus maniculatus* infected by Sin Nombre virus (Botten et al. 2000, Padula et al. 2004, Spengler et al. 2013). These similarities include virus persistence in solid tissues (particularly lung, heart, and kidney). However, in the study with *O. longicaudatus*, the Andes virus genome was found in the blood and kidneys of infected rodents but not in urine (Padula et al. 2004). In contrast, in the present study, we found that the RIOMV genome is excreted in faeces and urine, as well as organs and blood. These viral RNA loads confirm that the virus is produced in their organs as well as in faeces and urine. Additionally, RIOMV may have adapted to tissue culture, as this did not reduce its infectivity in*Sigmodontinae*. This finding is in contrast to reports of*Murinae* infected with Puumala virus, which was found to undergo genetic changes related to its adaptation in cell culture (Lundkvist et al. 1997).

Saliva is recognised as an important vehicle for the transmission of hantavirus among rodents, as they frequently bite each other. However, in the present study, RIOMV was not found in the saliva of infected animals. It is possible that excretion in saliva is initiated at a later stage of infection, as reported in experimental infections of*P. maniculatus* with Sin Nombre hantavirus, when substantial levels of viral RNA appeared only at day 14 ai (Botten et al. 2000).

Hantavirus infection in rodents other than primary hantavirus hosts (interspecies transmission) has also been reported, and these events are known as spillover (Jonsson et al. 2010). *N. lasiurus*or *Akodon sp* shown here are considered to be natural reservoirs of ARAQV (Suzuki et al. 2004, Figueiredo et al. 2009b). However, it was not possible to use ARAQV in our study. We chose to infect animals with RIOMV because it is adapted to tissue culture and allows for the production of suitable virus stocks (Bharadwaj et al. 1997). RIOMV has been reported to infect*Oligoryzomys microtis* rodents in Peru, Bolivia and Brazil (Figueiredo et al. 2014). This virus has been associated with two HCPS cases in Peru (Casapía et al. 2012), one case in French Guiana (Matheus et al. 2012) and another fatal case in Brazil (de Oliveira et al. 2014). Furthermore, we chose RIOMV because its phylogenetic clade has a N amino acid sequence that is 90% homologous with viruses of the Andes clade, including ARAQV (Souza & Figueiredo 2014).

The results shown here contribute to our understanding of viral kinetics in rodents and shed light on the mechanism of hantavirus transmission to humans by rodent excreta. Additionally, RIOMV can infect *Sigmodontinae* species other than*O. microtis*, the natural reservoir for RIOMV. Spillover events could allow for the natural transmission of RIOMV to these other rodent species.

Finally, though our findings are not generalisable to all *Sigmodontinae*and all hantaviruses, the results shown here reveal that hantavirus particles persist in both the urine and faeces of infected rodents for at least 18 days. This information is highly relevant to public health, as it is known that the inhaled products of excreta from infected animals can transmit hantavirus to humans and other animals. Therefore, our results corroborate the current notion that inhalation of excreta aerosols is the principal mechanism by which hantavirus is transmitted (Jonsson et al. 2010). However, further studies with different hantavirus strains, different routes and sites of infection, other rodent species and a larger number of animals per group are necessary to improve our understanding of hantavirus kinetics and its potential for transmission.
